# Tuboplasty as a reversal macrosurgery for tubal ligation, is pregnancy possible? A case series

**Published:** 2014-05

**Authors:** Fakhrolmolouk Yassaee

**Keywords:** *Tubal ligation*, *Reversal macro surgery*, *Rate of pregnancy*, *Fallopian tube reanastomoses*

## Abstract

**Background:** Reversal of tubal ligation is requested by some women for various reasons. The present study aims to determine the rate of pregnancy after tubal ligation reversal.

**Case: **In these case series, we reported fifteen women who requested tuboplasty after tubal ligation. In these cases reversal of tubal ligation was done in 15 women. Pregnancy occurred in 4 women (26.6%). Two term pregnancy and 2 abortions were determined.

**Conclusion:** The success rate of pregnancy after macro surgical reversal of tubal ligation is good and can be considered before in vitro fertilization. The type of tubal ligation and the procedure used will determine the best procedure for reversal and have a major impact on chance of success for reversal surgery.

## Introduction

One of the most effective methods for prevention of pregnancy is tubal ligation. This procedure is employed universally for limiting the size of the family. Tubal ligation is performed free of charge in Iran for women who choose to use this modality for family planning. Tubal ligation is usually done during cesarean section. Sometimes interval tubal ligation is done through mini laparotomy or laparoscopy. A tuboplasty is a surgical repair of the fallopian tubes, which carry the fertilized egg from the ovary to the uterus, often these tubes become damaged intentionally for the purpose of sterilization or unintentionally by previous infections, surgery, or scar tissue produced by endometriosis. The inside of the tube is very small, a little less than the diameter of a ball point pen cartridge in some areas. 

The fallopian tube is very complex structure. It not only functions as a conduit for the egg and the sperm to meet, but also provides nourishment for these germ cells during their journey. Often, a damaged portion of the tube can be removed and the healthy ends sewn back together. If the end of the tube is closed, it can be reopened and tied back in place. Each of these types of tuboplasty or repair has a different success rate. Success rates greatly depend on the age of the woman, the amount of the remaining tube and the technology used (1). There is a controversy about whether in vitro fertilization (IVF) or surgical reversal for tubal ligation are done when woman regrets after this procedure due to insufficient counseling. However, valid reasons for reversal surgery exist if the family loses their child or children in an accident, or if the woman marries again due to divorce in first marriage or due to death of her husband. These women may request reversal surgery after tubal ligation to be able to bear another child. There are some reports about reversal of tubal ligation (1, 2). 

In the study which is done by Jindal Promila and colleagues in India, the success rate of reversal of tubal ligation was none in patients with minilaparotomy for tubal ligation but there are no studies from Iran (2). The aim of this study was to report the success rate of reversal surgery for tubal ligation, (i.e. the rate of pregnancy) in 15 women who wanted to become pregnant after tubal ligation.

## Case report

We reported a case series of patients who requested tuboplasty after tubal ligation at Taleghani hospital affiliated to Shahid Beheshti University of Medical Sciences from April 1996 to April 2012. A history with special reference to reason for reversal and the date of tubal ligation was taken. In all the cases, semen analysis was done for their husbands to rule out azoospermia. 

Hysterosalpingography was done to know the site of ligation. All couples had written informed consent before surgery. The exclusion criteria were if history of fimbriectomy and azoospermia. Women age was between 30-40 years old. Reasons for tuboplasty are presented in table I, and type of tubal ligation reversal and success rate is presented in table II.

Case 1: A 30 years old with 3 children. Tubal ligation was done. All her 3 children died by carbon monoxide event in the bathroom. So she requested tuboplasty. It was done in 1996.

Case 2: A 30 years old with 3 children. Tubal ligation was done on 1995. She requested tuboplasty due to second marriage and it was done in 1998. She became pregnant and delivered a term fetus.

Case 3: A 32 years old with 2 children. Tubal ligation was done in 2001. She requested tuboplasty due to death of her neonate 1 month after tubal ligation. So tuboplasty was done in 2001. After 3 years she became pregnant but abortion occurred. Again she became pregnant in 2011 but fetal demise followed by abortion.

Case 4: A 33 years old with 2 children. Tubal ligation was done during last cesarean section on 2000. After death of her son she requested tuboplasty. So it was done in 2003. She did not become pregnant.

Case 5: A 39 years old with 3 children. Tubal ligation was done 1996.She had second marriage. So she requested tuboplasty and it was done in 2004.

Case 6: A 32 years old with 3 children. Tubal ligation was done on 2003. After death of one of her children she requested tuboplasty. It was done in 2004.

Case 7: A 31 years old with 2 children. Tubal ligation was done during cesarean section on 2004. After death of her child she requested tuboplasty. It was done in 2004, 5 months after tubal ligation.

Case 8: A 32 years old with 2 children. Tubal ligation was done during last cesarean section in 2004. She had second marriage due to death of her husband. So she requested tuboplasty, and it was done in 2006. She did not become pregnant.

Case 9: A 32 years old with 2 children. Tubal ligation was done during last cesarean section in 2001. She regretted and requested tuboplasty. It was done in 2006.

Case 10: A 34 years old with 3 children and one abortion. Tubal ligation was done during last cesarean section on 2006. After 4 months she regretted and became anxious. So she requested tuboplasty and it was done in 2006.

Case 11: A 40 years old with 2 children. Tubal ligation was done during last cesarean section in 1994. She requested tuboplasty due to second marriage and it is done on 2006. She became pregnant but abortion occurred.

Case 12: A 31 years old with 1 child. Tubal ligation was done in 2001. She regretted and requested tuboplasty. It was done in 2009.

Case 13: A 40 years old with 1 child. Tubal ligation was done in 2006 due to unknown reason (she did not know). After 4 years she requested tuboplasty and it was done in 2010.She became pregnant in 2011 but she aborted it.

Case 14: A 38 years old with 3 children. Tubal ligation was done during last cesarean section in 2003. After death of her 2 children and failure of in vitro fertilization she requested tuboplasty. It is done in 2011. She did not become pregnant.

Case 15: A 30 years old with 2 children. Tubal ligation was done in 2003. Due to second marriage she requested tuboplasty and it was done in 2012.

**Table I:** Reasons for Tuboplasty

**Reason**	**Number**	**Percent**
Death of 3 children	1	6.6
Death of 2 children	1	6.6
Death of male child	1	6.6
Death of 1 child	3	20
Tubal ligation done without counseling	1	6.6
Second marriage	5	33
Regret for tubal ligation	3	20
Total	15	100

**Table II:** Type of tubal ligation reversal and success rate

**Type of tubal Anastomosis**	**Total **	**Term pregnancy**	**Abortion **	**Lost to follow up**
Bilateral isthmic isthmic	8 (53.5)	2 (25)	2 (25)	4 (50)
Bilateral isthmic ampullary	1(6.6)	**-**	**-**	1(6.6)
Isthmic isthmic +ampuloinfudibular	3 (20)	**-**	**-**	3 (20)
Bilateral ampulo infundibular	3 (20)	**-**	**-**	3 (20)
Total	15(100)			11 (73.3)

Data are presented as n (%).

## Discussion

In our study the success rate of reversal surgery for tubal ligation was 26.6%. During reproductive life, the need for reversal of tubal ligation may arise (3-15). Death of one or more children has been reported as the most reason for reversal of tubal ligation (2). In our study death of the male child was the most reason for reversal of tubal ligation. The technique of tubal ligation is an important factor affecting the rate of success in reversal surgery. Because diameter of the fallopian tubes varies from one end to the other, the best chance for success occurs when the diameter of the two remaining section of the fallopian tubes are almost identical. 

In cases where the two remaining ends of the tubes are of different diameter (for example, a narrow end of the tube close to the uterus is being connected to a wider end near the end of the fallopian tube) success rates for pregnancy are lower (4). Isthmic-isthmic anastomosis is the simplest type of anastomosis as the lumina are comparable in size (3, 5, 9). In our study, all 4 women who conceived had isthmic-isthmic anastomosis so the chance for reversal of tubal ligation is comparable with the success rate for IVF. Moreover, IVF has to be repeated for each pregnancy but tuboplasty is life-long. The length of the tube after tuboplasty is important for success. So the ideal candidate for tubal ligation reversal is a woman who has nearly equal diameter of the remaining ends of the tubal sections, and those whose tubes are at least three to four inches long following reversal of the tubal ligation as the fallopian tubes are approximately 8 inches long before tubal ligation (6). 

In our study all the 4 women who became pregnant their length of tubes was more than 4 inches after tuboplasty. Overall, success rates for tubal ligation reversal can vary from 20-70% (4, 8). Sometimes tubal ligation reversal is desired not for the purpose of having children, but to reverse the effects of post tubal ligation syndrome such as symptoms of menopause, anxiety, or irregular heavy periods experienced by women (4). In our study one of our patients requested tubal ligation reversal due to anxiety and mood disorder such that she was prescribed antidepressant drugs. Eleven women were lost to follow up; some of them might have got pregnant. This study is still continuing in a prospective manner and we hope to find the cases lost to follow-up. This is the first report of success in tubal ligation reversal in Iran. 

## Conclusion

We recommend to our colleagues to have special attention to the size of the fallopian tubes which is removed during tubal ligation. Tubal ligations done at the isthmus have the best chance of success for reversal. Sufficient counseling before tubal ligation is mandatory. 
